# Validity of Ultra-Short-Term Heart Rate Variability Derived from Femoral Arterial Pulse Waveform in a British Military Cohort

**DOI:** 10.1007/s10484-024-09652-3

**Published:** 2024-07-11

**Authors:** Rabeea Maqsood, Susie Schofield, Alexander N. Bennett, Ahmed Khattab, Anthony M. J. Bull, Nicola T. Fear, Christopher J. Boos

**Affiliations:** 1 Department of Medical Sciences and Public Health, Faculty of Health & Social Sciences, Bournemouth University, Bournemouth, BH8 8GP UK; 2 National Heart and Lung Institute, Faculty of Medicine, Imperial College London, London, SW3 6LR UK; 3 Academic Department of Military Rehabilitation, Defence Medical Rehabilitation Centre, Stanford Hall Estate, Loughborough, LE12 5QW UK; 4 Centre for Injury Studies, Department of Bioengineering, Imperial College London, London, SW7 2AZ UK; 5 Academic Department of Military Mental Health and King’s Centre for Military Health Research, King’s College London, London, SE5 9RJ UK; 6 Department of Cardiology, University Hospitals Dorset, Poole Hospital, Poole, Poole, BH15 2JB UK

**Keywords:** RMSSD, Validity, Ultra Short term, Military, Parasympathetic tone

## Abstract

Various non-electrocardiogram (ECG) based methods are considered reliable sources of heart rate variability (HRV) measurement. However, the ultra-short recording of a femoral arterial waveform has never been validated against the gold-standard ECG-based 300s HRV and was the aim of this study.

A validity study was conducted using a sample from the first follow-up of the longitudinal ADVANCE study UK. The participants were adult servicemen (*n* = 100); similar in age, rank, and deployment period (Afghanistan 2003–2014). The femoral arterial waveforms (14s) from the pulse wave velocity (PWV) assessment, and ECG (300s) were recorded at rest in the supine position using the Vicorder™ and Bittium Faros™ devices, respectively, in the same session. HRV analysis was performed using Kubios Premium. Resting heart rate (HR) and root mean square of successive differences (RMSSD) were reported. The Bland-Altman %plots were constructed to explore the PWV-ECG agreement in HRV measurement. A further exploratory analysis was conducted across methods and durations.

The participants’ mean age was 38.0 *±* 5.3 years. Both PWV-derived HR (*r* = 0.85) and RMSSD (r_s_=0.84) showed strong correlations with their 300s-ECG counterparts (*p* < 0.001). Mean HR was significantly higher with ECG than PWV (mean bias: -12.71 *±* 7.73%, 95%CI: -14.25%, -11.18%). In contrast, the difference in RMSSD between the two methods was non-significant [mean bias: -2.90 *±* 37.82% (95%CI: -10.40%, 4.60%)] indicating good agreement. An exploratory analysis of 14s ECG-vs-300s ECG measurement revealed strong agreement in both RMSSD and HR.

The 14s PWV-derived RMSSD strongly agrees with the gold-standard (300s-ECG-based) RMSSD at rest. Conversely, HR appears method sensitive.

**Supplementary Information:**

The online version contains supplementary material available at 10.1007/s10484-024-09652-3.

## Introduction

Heart rate variability (HRV) refers to the fluctuation in the time interval between consecutive heartbeats (Malik, 1996) and provides an objective index of overall physical and mental health (Kemp & Quintana, 2013). The RR intervals (RRi) required for HRV measurement have been traditionally acquired using electrocardiogram (ECG) recordings over long (24 h) and short (5–10 min) periods (Shaffer & Ginsberg, 2017). The analysis of recordings shorter than 5 min is referred to as ultra-short-term HRV (HRV_UST_) analysis (Shaffer et al., 2020). The clinical utility of short-term HRV has increased over the last decade, especially in the field of cardiovascular research given the association between lower HRV and elevated cardiovascular risk (Fang et al., 2020; Schuster et al., 2016). Owing to its short signal acquisition time and ease of measurement, studies have shown a huge potential of HRV_UST_ as a cardiovascular risk measure (Orini et al., 2023). However, there is a need for robust validation of HRV_UST_ prior to its wider use.

There is a plethora of studies that have investigated the agreement between pulse waveform-derived HRV with traditional ECG-based HRV measurement using the cardiac inter-beat interval. This has been mainly assessed using the radial and brachial arterial signals. Examples include ithlete™ (Flatt & Esco, 2013), the wearable Bora Band™ wristband (Taoum et al., 2022), the Uscom™ BP + device (Boos et al., 2022), NIVA band™ and the AD Lab Instrument^®^ (Kumar e al., 2023) in healthy adults (Hernando et al., 2018), and Portapres™ in a patient population (Munoz et al., 2015). We have also previously reported the reliability of femoral arterial waveforms for HRV_UST_ measurement using the Vicorder™ device (Skidmore Medical Limited, Bristol, UK) during pulse wave velocity (PWV) assessment among British servicemen with combat injuries (Maqsood et al., 2023a). However, its agreement with the gold-standard 300s electrocardiogram (ECG) derived HRV has not been investigated.

In this study, we sought to assess the validity of femoral arterial waveform-derived HRV_UST_ with that of standard ECG-derived short-term HRV. We hypothesised that pulse waveform-derived- HRV_UST_ (14s) would offer acceptable agreement and strongly correlate with the gold standard 300s ECG-related short-term (300s) HRV.

## Methods

### Study Design

This was a validity study in which femoral arterial pulse waveform-derived HRV_UST_ was compared to traditional 300s ECG-based HRV measured in the same session. Validity or agreement refers to the extent to which a “new method” corresponds to the “criterion” (Kottner &Streiner, 2011). The study population consisted of 100 participants who attended their first follow-up in the ongoing ArmeD serVices trAuma and rehabilitatioN outComE (ADVANCE) Cohort study. The full protocol of the ADVANCE study can be accessed elsewhere (Bennett et al., 2020).

The ADVANCE study has been approved by the UK Ministry of Defence Research Ethics Committee (MoDREC) (protocol number 357/PPE/12) and is conducted in compliance with the Declaration of Helsinki (Bennett et al., 2020). Complying with data-confidentiality terms, the authors had no access to participants’ identity during and after data collection. All participants in this study took part voluntarily and provided full informed and written consent. The data for the present study were collected between January 2020 and December 2022. All assessments were conducted by trained research nurses at the Defence Medical Rehabilitation Centre (DMRC), Stanford Hall, Loughborough (Bennett et al., 2020).

### Participants

The sample consisted of a subset of 100 servicemen, recruited into the ongoing ADVANCE study. The participants were randomly selected from the cohort (*n* = 1053). Of 100, 50 participants sustained a serious physical combat injury (e.g., burns, fractures, amputation) during their deployment to Afghanistan on Operation HERRICK (2003–2014) whereas 50 were uninjured during the same deployment. None of the participants were on beta-blockers at the time of the present study. Participants did not have a history of cardiovascular, renal, or liver disease prior to recruitment.

### HRV Measure

The root mean square of successive differences (RMSSD) was used to measure HRV_UST_ and short-term HRV. Resting heart rate (HR) was also reported. The inclusion of resting RMSSD was informed by previous research, which established its reliability and validity in HRV_UST_ and short-term analysis (Munoz et al., 2015; Nussinovitch et al., 2011; Thong et al., 2003). Furthermore, the RMSSD is also a known marker of parasympathetic modulation and remains uninfluenced by respiration rate (Laborde et al., 2017). This further increases its applicability in clinical research.

### Data Collection

All data were collected during the daytime at Stanford Hall by a trained research nurse. The participants were asked to lie down in the supine position and relax. All participants were requested to fast for at least 8 h before the assessments. The average room temperature was 21 degrees Celsius. The Vicorder™ (Skidmore Medical Limited, Bristol, UK) and the Bittium Faros™ (Mega Motion Faros 180 recorder: Mega Electronics Ltd., Pioneerinkatu, Finland) devices were set up on the participants simultaneously for data collection in the same session. Participants were asked to breathe normally throughout the recordings (Bennett et al., 2020).

A plethysmography-based sensor (30 mm) was placed over the neck, and a wide cuff was placed over the left thigh to acquire carotid and femoral pulse waveforms, respectively. The use of the Vicorder™ has been validated for pulse wave analysis previously (Pucci et al., 2013). All recordings were taken in triplicates, ipsilaterally unless not possible due to amputation (*n* = 12) in injured participants. The recording (waveform) with the greatest number of beats was exported using the Vicorder™ software for offline analysis of HRV_UST,_ as per our previous work (Maqsood et al., 2023a).

The Bittium Faros™ device was used to acquire ECG signals for HRV measurement. Surface ECG electrodes were placed under the right and left clavicle and a third over the lower left rib frame. The electrodes were connected to the Bittium Faros™ device. The sampling rate was set at 250 Hz. ECG data collection started simultaneously with the Vicorder™ assessment and lasted for about 15–20 min on average, with the last 10 min comprising 5 min of spontaneous followed by 5 min of paced breathing protocol.

### HRV Analysis Protocol

For both HRV_UST_ and short-term HRV analysis, Kubios premium (V.3.2, The Biomedical Signals Analysis and Medical Imaging Group, University of Kuopio, Finland) was used. The following operations were set constant for both ultra- and short-term analysis of HRV recordings in Kubios premium: the smoothness priors (500), and interpolation (cubic spline: 4 Hz with 50ms R-R threshold) as previously used (Canino et al., 2022). All signals were visually inspected for ectopic and erroneous beats and manually corrected wherever needed. The data were analysed by a single analyst (RM) to avoid bias.

For HRV_UST_, only signals from femoral arterial waveforms (14s) were used to derive RMSSD, based on its proven superior reliability compared with carotid arterial waveforms (Maqsood et al., 2023a). The automatic correction and noise levels were set to none and medium, respectively throughout the analysis. The analysis type was set at single except for shorter recordings (< 10s), where the ‘merge’ type was applied.

For short-term HRV analysis of RMSSD, a 300s recording of ECG under spontaneous breathing protocol was analysed. This analysis window was estimated based on when the PWV recording overlapped with that of the ECG as a proxy for simultaneous data collection. The correction was set to automatic with medium noise in Kubios.

### Sample Size Calculation

In a previous study of 70 healthy adults, Nussinovitch and colleagues examined comparative RMSSD scores for 60s versus 300s ECG recordings (Nussinovitch et al., 2011). They observed a paired mean difference of 2ms. Based on this previous work and our own published work using PWV-based HRV_UST_ (Maqsood et al., 2023b), we estimated that a sample size of at least 85 paired HRV_UST_ versus 300s HRV readings would have at least 80% power to detect a paired difference in RMSSD of *≤* 3ms at a two-sided alpha of 0.05.

### Statistical Analysis

The results were presented as mean ± standard deviation (SD), median and interquartile (IQR), or number and percentage. Data were assessed for normality via visual inspection of histograms and QNorm plots. Paired *t*-test and Wilcoxon signed-rank test were used for parametric and non-parametric comparisons, respectively. Pearson’s correlation coefficient (*r*) and Spearman’s rho (r_s_) were calculated for parametric and non-parametric assessment of correlation, respectively. The strength of the relationship was interpreted as weak (0.10.-0.39), moderate (0.40–0.69), strong (0.70–0.89), and very strong (0.90–1.00) (Schober et al., 2018). The data were inspected for outliers in GraphPad Prism (GraphPad Software, LLC) using the RObust regression and oUTlier (ROUT) removal method (Motulsky & Brown, 2006). The detected outliers were found to be true outliers and were excluded in the sensitivity analysis (14s PWV vs. 300s ECG HR *n* = 1, RMSSD *n* = 2).

We additionally undertook a further analysis to explore the influence of change in method and durations on the agreement. This was done by analysing data from different methods but the same duration (14s PWV vs. 14s ECG), and different durations but the same method (14s ECG versus 300s ECG).

As correlation does not imply agreement (Ranganathan et al., 2017), the agreement between the new method (PWV) and the criterion (ECG) was assessed using the Bland-Altman analysis (Bland & Altman, 1986). Heteroscedasticity was observed for the difference in absolute RMSSD. Given this, the percent difference plot with the Bland-Altman analysis was plotted using the absolute values as recommended (Dewitte et al., 2002). The mean bias was defined as the average difference in RMSSD between PWV and ECG sources (reported in %). The upper and lower limits of agreement (*±* LoA) with a 95% confidence interval (CI) were also reported (in %). The Bland-Altman analysis was performed for 14s-PWV vs. 300s-ECG-based HR and RMSSD (with and without outliers for sensitivity analysis). All statistical analyses were performed using Stata (V 17.0; StataCorp LLC) unless stated otherwise.

## Results

The present analysis included 100 participants (50 injured and 50 uninjured) who were similar in demographic, anthropometric, and lifestyle characteristics (Table 1). Of the 50 injured participants, 12 were amputees. The mean age of the participants at the time of their first follow-up visit was 38.0 *±* 5.3 years. The majority of participants were White (91%) and had Junior rank (69%). Most of the participants were non-smokers (54%) (Table 1).

Resting HR was significantly lower with PWV than the ECG-derived HR (mean difference: − 7.13 *±* 4.36, *p* < 0.001), respectively. The median and mean differences in RMSSD between the two methods were statistically non-significant (Table 2). These differences also remained statistically non-significant with the exclusion of outliers in the sensitivity analyses (supplementary information, Table S1).

**Fig. 1:**
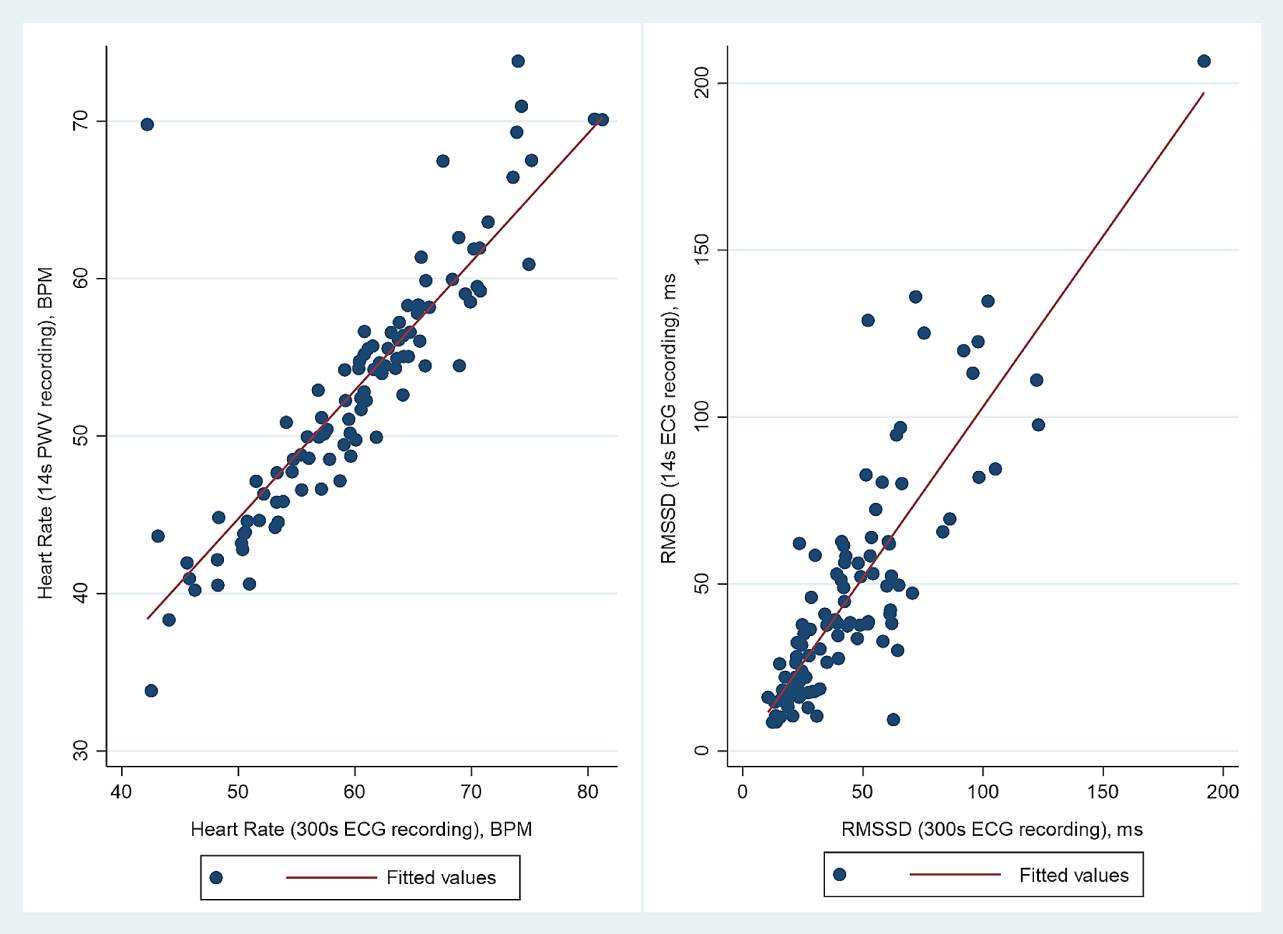
Scatter plot of the association between HRV scores from two different methods (14s PWV vs. 300s ECG). Notes: PWV, Pulse Wave Velocity; ECG, electrocardiogram; BPM, Beats Per Minute; ms, millisecond; HR, Heart Rate; RMSSD, Root Mean Square of Successive Differences

**Fig. 2:**
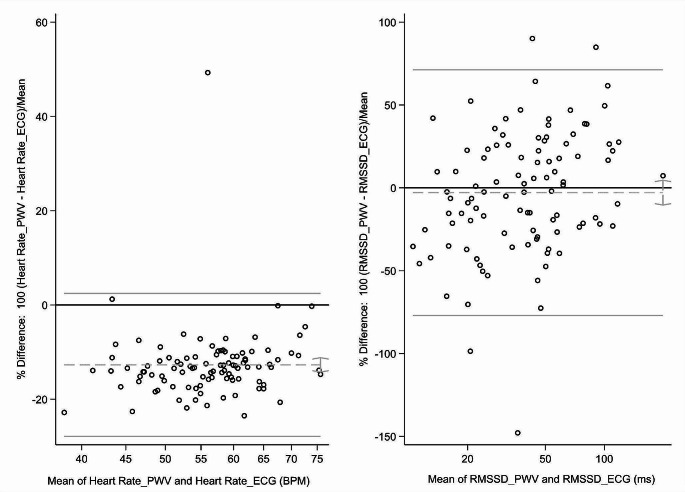
The Bland-Altman Percent Plot for resting HR and RMSSD values from the new method and gold-standard methods (14s PWV vs. 300s ECG). Notes: PWV, Pulse Wave Velocity; ECG, electrocardiogram; BPM, Beats Per Minute; ms, millisecond; HR, Heart Rate; RMSSD, Root Mean Square of Successive Differences. Absolute values have been used in the Bland-Altman percent plots. The x-axis represents the mean HR and RMSSD from PWV and ECG (PWV + ECG/2), and the y-axis represents the percentage of the difference in HRV index between PWV and ECG (100* PWV-ECG)/mean. Grey dotted lines denote mean bias (%), and grey solid lines are 95% confidence intervals of bias (lower and upper limits of agreements)

Resting HR (*r* = 0.85) and RMSSD (r_s_= 0.82) from a 14s PWV recording strongly correlated with their 300s ECG-derived counterparts (*p* < 0.001) (Table 2; Fig. 1). The Bland-Altman analysis showed a good agreement in RMSSD scores between 14s PWV vs. 300s ECG data (mean bias: -2.90 *±* 37.82%, 95%CI mean%difference: -10.40%, 4.60%). In contrast, a poor agreement was observed for 14s PWV derived HR vs. 300s ECG counterpart (mean bias: -12.71 *±* 7.73% 95%CI mean%difference: -14.25%, -11.18%) (Fig. 2).

The sensitivity analysis without the outliers showed similar results with the same trends. The mean bias for HR was − 13.34 *±* 4.56% (95%CI mean%difference: -14.25%, -12.43%) and for RMSSD: -4.45 *±* 36.53% (95%CI mean%difference: -11.78%, 2.86%) (supplementary information, Fig S1).

**Table 1:** Demographic characteristics of the sample

	Participants (*n* = 100)
Age at first follow-up assessment, years	38.09 *±* 5.30
Ethnicity	
-White -Other	91 (91%) 9 (9%)
Rank	
-Junior rank -Senior Rank -Commissioned Officers	69 (69%) 22 (22%) 9 (9%)
Smoking status	
-Ex-smoker -Never -Smoker	27 (27%) 54 (54%) 19 (19%)
Height, cm	178.4 *±* 6.47
Weight, kg	90.81 *±* 15.58

Notes: Data are shown as mean and standard deviation or number (percentage)

**Table 2:** Comparison of HRV values from the new method (14s PWV) with the gold standard (300s ECG) (*n* = 100)

	PWV (14s)	ECG (300s)	Mean Difference *±* SD	Correlation* (95% CI) (*p*-value)
HR, BPM	53.13 *±* 7.97	60.27 *±* 8.39	-7.13 *±* 4.36 (*p* < 0.001)	0.85 (0.79–0.90) (*p* < 0.001)
RMSSD, ms	47.63 *±* 35.53	45.75 *±* 29.36	1.87 *±* 19.01 (*p* = 0.32)	0.84 (0.77–0.89) (*p* < 0.001)
RMSSD, ms (median, IQR)	38.16 (20.49, 61.81)	40.35 (23.49, 60.77)	-0.79 (-10.43, 10.43) (*p* = 0.78)	0.82 (0.74–0.87) (*p* < 0.001)

Notes

Data are shown as mean and standard deviation or median and interquartile range (IQR)

PWV, Pulse Wave Velocity; ECG, electrocardiogram; BPM, Beats Per Minute; ms, millisecond; HR, Heart Rate; RMSSD, Root Mean Square of Successive Differences

*Pearson’s (*r*) for parametric and Spearman’s rho (r_s_) for non-parametric correlation.

The exploratory comparison revealed that the mean and median differences in RMSSD remained non-significant regardless of the difference in methods and duration of recording (Table 3). However, the mean difference between the 14s ECG-derived HR vs. 300s ECG-derived HR was smaller and non-significant (-0.23 *±* 2.85 BPM, *p* = 0.40) (Table 3).

**Table 3:** Comparison of HRV values by method and length of recording (*n* = 100)

	PWV (14s) A	ECG (14s) B	ECG (300s) C	Δ A-B (14s PWV vs. 14s ECG)	Δ B-C (14s ECG vs. 300s ECG)	Δ A-C (14s PWV vs. 300s ECG)
HR, BPM	53.13 *±* 7.97	60.03 *±* 9.15	60.27 *±* 8.39	-6.90 *±* 5.02 (*p* < 0.001)	-0.23 *±* 2.85 (*p* = 0.40)	-7.13 *±* 4.36 (*p* < 0.001)
RMSSD, ms	47.63 *±* 35.53	43.53 *±* 29.95	45.75 *±* 29.36	4.09 *±* 26.59 (*p* = 0.12)	-2.22 *±* 20.18 (*p* = 0.27)	1.87 *±* 19.01 (*p* = 0.32)
RMSSD, ms (median, IQR)	38.16 (20.49,61.81)	34.62 (21.58,58.01)	40.35 (23.49, 60.77)	0.52 (-10.19, 11.31) (*p* = 0.54)	-1.01 (-7.41, 5.26) (*p* = 0.37)	-0.79 (-10.43, 10.43) (*p* = 0.78)

Notes

Data are shown as mean and standard deviation or median and interquartile range (IQR)

PWV, Pulse Wave Velocity; ECG, electrocardiogram; BPM, Beats Per Minute; ms, millisecond; HR, Heart Rate; RMSSD, Root Mean Square of Successive Differences

## Discussion

This validity study aimed to compare RMSSD from a 14s PWV recording with that of a 300s ECG recording in a sample from a military cohort. The findings support our hypothesis of a strong positive correlation and agreement in RMSSD between the new and criterion methods. In contrast, despite a strong correlation, a good agreement was not observed in HR which was significantly lower in the 14s PWV versus the 300s ECG method of calculation.

To the authors’ knowledge, this is the first study to have examined the validity of a PWV-derived RMSSD using the Vicorder™ device with that using the 300s continuous ECG recording. Given the heterogeneity in HRV measurement device, length of HRV_UST_ recording, the population, and reporting of mean bias in varied units, the findings of our research may not be directly comparable with previous studies. However, a strong correlation and acceptable agreement between the two methods may be compared. For example, Boos and colleagues reported higher RMSSD values with a shorter (10s) PPG-based method than the 300s ECG recording with a median difference of -2.3ms in healthy military servicemen and women (Boos et al., 2022). In contrast, Munoz et al. examined the agreement between RMSSD measured using PPG versus ECG recordings over multiple lengths and reported that RMSSD values increased with the recording length. They reported a mean bias of 0.08ms and a correlation coefficient of 0.94 (95%CI 0.93–0.94) in their comparison of average 10s PPG versus a 300s ECG recording in a mixed-gender patient population (Munoz et al., 2015). However, comparison with other non-ECG versus ECG-based method studies is limited given the difference in duration of HRV_UST_ recording (e.g. >1 min) (Kumar et al., 2023; Taoum et al., 2022).

The comparison presented in this study was not merely based on the method (PWV versus ECG) but also on the duration of the recording (14s versus 300s). The difference in RMSSD remained non-significant across methods (PWV vs. ECG) and durations (14s, 300s) of recording. This indicates that the RMSSD (as a measure of HRV) may offer more applicability in clinical research given its reliability and validity across the devices than HR alone. This is because resting HR measurement appears to be more sensitive to the ECG-based method, demonstrating more consistency and agreement than the PWV-derived measurement regardless of the duration of the recording. This is in contrast to what has been previously reported i.e. a mean difference of 0.18 ± 11.41 BPM in a PPG vs. ECG-based HR measurement (Boos et al., 2022). Other relevant PPG vs. ECG-based studies have not reported the agreement for HR (Flatt & Esco, 2013; Munoz et al., 2015). It is important to note that the HR reported in this study was calculated from the 14s femoral arterial waveform by Kubios, and not from the “crude” PWV output for consistency. The difference in HR agreement may be explained by the difference in sampling frequency for ECG (250 Hz) vs. pulse waveform data (556 Hz) as discussed elsewhere (Taoum et al., 2022). The guidance on sampling frequency using the neuroimaging methods to derive valid HRV measures exists (Burma et al., 2021); however, clinical populations are under-represented in such studies. Therefore, the topic warrants further exploration for accurate comparison.

The first strength of this analysis is its uniqueness in validating the use of femoral arterial waveform that captures the “actual” short-term HRV measurement. Second, the majority of the data were of excellent quality. On average, only 0.13% of beats were corrected on the ECG recordings via automatic correction supplemented with visual inspection. This enabled the use of continuous segments for the calculation of successive differences for RMSSD analysis as previously described (Munoz et al., 2015). Third, a standardized protocol was followed for PWV and HRV data collection. Last, all data were analysed by a single data analyst (RM) minimising the subjective bias.

The presented findings should be interpreted under the context of some limitations. The selection of the overlapping segment on the ECG recording was based on estimation and not on the actual timestamp of when the PWV was recorded. Our sample consisted of both injured and uninjured participants; whether combat injury affects the presented agreement could not be determined due to the smaller sample size and was beyond the scope of the study. Only one time-domain measure of HRV (RMSSD) was validated in this study, limited by its shorter but reliable recording length of 14s, as opposed to frequency and non-linear measures, which would have required longer recording (Malik, 1996; Munoz et al., 2015). While the RMSSD values derived from the pulse waveform agree with those derived from the ECG waveform in this study, some evidence suggests that pulse rate variability (derived from photoplethysmography) should not be considered a surrogate for HRV (Burma et al., 2024; Mejía-Mejía et al., 2020). The possibility of age and arterial stiffness affecting the arterial waveforms, such as cerebral arterial waveform (Lefferts et al., 2020) should also be kept in context when interpreting our findings. This is because our sample partly consisted of those with CRTI who have been reported to have a higher risk of arterial stiffness than their uninjured counterparts (Boos et al., 2021). Lastly, our findings may be generalisable to only male military veterans and personnel or populations with similar characteristics to our study population.

The short-term (300s) ECG-based measurement of HRV has been considered a gold-standard method for studying heart rate dynamics (Malik, 1996). Within this scope, the acceptable agreement between the 14s PWV and 300s ECG-based RMSSD offers implications for the extended use of the Vicorder device beyond PWV measurement. This is an important practical implication, especially where access to ECG recordings is difficult. This might also be considered a time-saving and cost-effective alternative to the expensive ECG recording devices. Given the promising performance of RMSSD in our validity study, the use of ultra-short term RMSSD in a PPG or ECG-based wearable device may be considered for HRV tracking in a military population or other populations with similar characteristics.

## Conclusion

RMSSD derived from a 14s recording of femoral arterial waveform offers validity and agrees with the 300s ECG-derived RMSSD at rest in a military cohort. While measurement of the resting heart rate may be method sensitive, RMSSD seems unaffected by the difference in method and durations of the recording in our sample.

## Electronic Supplementary Material

Below is the link to the electronic supplementary material.

Supplementary Material 1: Table S1: Sensitivity analysis of HRV values from the new method (14s PWV) in comparison with the gold standard method (300s ECG) without the outliers; Table S2: Sensitivity analysis of HRV comparison across methods and durations of recording without the outliers; Fig S1: The sensitivity analysis of the Bland-Altman Percent Plots for resting HR and RMSSD values from the new method and gold-standard (14s PWV vs. 300s ECG) without outliers.

## Data Availability

Data may be obtained from a third party and are not publicly available. Only the authorised authors (R.M, C.J.B) had access to the data of this study. Given the sensitive nature of the participants, data have not been made widely available. Requests for data will be considered on a case-by-case basis and subject to the UK Ministry of Defence clearance. More information can be found at: https://www.advancestudydmrc.org.uk/.
