# Supplementary material for: Validity of Ultra-Short-Term Heart Rate Variability Derived from Femoral Arterial Pulse Waveform in a British Military Cohort
Source: Appl Psychophysiol Biofeedback. 2024 Jul 11;49(4):619–27. doi: 10.1007/s10484-024-09652-3 (PMC11588943; doi:10.1007/s10484-024-09652-3)
Supplement: Supplementary file 1 — Supplementary Material 1: Table S1: Sensitivity analysis of HRV values from the new method (14s PWV) in comparison with the gold standard method (300s ECG) without the outliers; Table S2: Sensitivity analysis of HRV comparison across methods and durations of recording without the outliers; Fig S1: The sensitivity analysis of the Bland-Altman Percent Plots for resting HR and RMSSD values from the new method and gold-standard (14s PWV vs. 300s ECG) without outliers. [file 10484_2024_9652_MOESM1_ESM.docx]

**Validity of ultra-short term heart rate variability derived from femoral arterial pulse waveform in a British military cohort.**

Rabeea Maqsood*^1,2^, Susie Schofield^2^, Alexander N Bennett^2,3^, Ahmed Khattab^1^, Anthony M J Bull^4^, Nicola T Fear^5^, Christopher J Boos^1,6^, for the ADVANCE Study

^1^Department of Medical Sciences and Public Health, Faculty of Health & Social Sciences, Bournemouth University, BH8 8GP, Bournemouth, United Kingdom.

^2^National Heart and Lung Institute, Faculty of Medicine, Imperial College London, SW3 6LR, London, United Kingdom.

^3^Academic Department of Military Rehabilitation, Defence Medical Rehabilitation Centre, Stanford Hall Estate, LE12 5QW, Loughborough, United Kingdom.

^4^Centre for Injury Studies, Department of Bioengineering, Imperial College London, SW7 2AZ, London, United Kingdom.

^5^Academic Department of Military Mental Health and King’s Centre for Military Health Research, King’s College London, SE5 9RJ, London, United Kingdom.

^6^Department of Cardiology, University Hospitals Dorset, Poole Hospital, Poole, BH15 2JB, Poole, United Kingdom.

**Corresponding author*:** Rabeea Maqsood; Faculty of Health & Social Sciences, Bournemouth University, Bournemouth, BH8 8GP

**Email:** [rmaqsood@bournemouth.ac.uk](mailto:rmaqsood@bournemouth.ac.uk)

**Orcid id: 0000-0002-8551-0442**

# **Supplementary information**

Table S1: Sensitivity analysis of HRV values from the new method (14s PWV) in comparison with the gold standard method (300s ECG) without the outliers.

Table S2: Sensitivity analysis of HRV comparison across methods and durations of recording without the outliers.

Fig S1: The sensitivity analysis of the Bland-Altman Percent Plots for resting HR and RMSSD values from the new method and gold-standard (14s PWV vs 300s ECG) without outliers.

**Supplementary Information**

**Table S1: Sensitivity analysis of HRV values from the new method (14s PWV) in comparison with the gold standard method (300s ECG) without the outliers**

|  | **PWV (14s)** | **ECG (300s)** | **Mean Difference+SD** | **Correlation***  **(95%CI)**  **(*p*-value)** |
| --- | --- | --- | --- | --- |
| HR^†^, BPM | 52.96+ 7.83 | 60.45+8.23 | -7.48+2.60  (p<0.001) | 0.94  (0.92-0.96)  (*p*<0.001) |
| RMSSD^‡^, ms | 45.89+33.71 | 45.42+29.53 | 0.47+16.40  (p=0.77) | 0.87  (0.81-0.91)  (*p*<0.001) |
| RMSSD^‡^, ms  (median, IQR) | 37.95  (20.26, 58.63) | 39.69  (23.44, 60.54) | -1.02  (-10.58, 9.99)  (p=0.94) | 0.82  (0.74-0.87)  (*p*<0.001) |

**Notes:**

-Data are shown as mean and standard deviation or median and interquartile range.

-PWV, Pulse Wave Velocity; ECG, electrocardiogram; BPM, Beats Per Minute; ms, millisecond; HR, Heart Rate; RMSSD, Root Mean Square of Successive Differences

-*Pearson’s (*r)* for parametric and Spearman’s rho (r_s_) for non-parametric correlation.

^-^†n=99.

-‡n=98.

**Fig S1: The sensitivity analysis of the Bland-Altman Percent Plots for resting HR and RMSSD values from the new method and gold-standard (14s PWV vs 300s ECG) without outliers**

**Notes:**

-PWV, pulse wave velocity; ECG, electrocardiogram; BPM, Beats Per Minute; ms, millisecond; HR, Heart Rate; RMSSD, Root Mean Square of Successive Differences

-Absolute values have been used in the Bland-Altman percent plots. The x-axis represents the mean HR† and RMSSD‡ from PWV and ECG (PWV+ECG/2), and the y-axis represents the percentage of the difference in HRV index between PWV and ECG (100* PWV-ECG)/Mean.

-Grey dotted lines denote mean bias (%), and grey solid lines are 95% confidence intervals of bias (lower and upper limits of agreements).

^-^†n=99, ‡n=98.

|  | **(14s PWV vs 14sECG)** | **(14s ECG vs 300s ECG)** | **(14s PWV vs 300s ECG)** |
| --- | --- | --- | --- |
| HR^†^, BPM | -7.07+3.24  (p<0.001) † | -0.51+1.96  (p=0.01) † | -7.48+2.60  (*p*<0.001) ‡ |
| RMSSD^‡^, ms | 1.69+23.02  (p=0.46) ** | -1.04+14.20  (p=0.47) * | 0.47+16.40  (*p*=0.77) † |
| RMSSD^‡^, ms  (median, IQR) | 0.08  (-10.85, 10.85)  (p=0.91) ** | -0.62  (- 5.55, 5.17)  (p=0.43) * | -1.02  (-10.58, 9.99)  (*p*=0.94) † |

**Table S3: Sensitivity analysis of HRV comparison across methods and durations of recording without the outliers**

**Notes:**

-Data are shown as mean and standard deviation or median and interquartile range.

-PWV, Pulse Wave Velocity; ECG, electrocardiogram; BPM, Beats Per Minute; ms, millisecond; HR, Heart Rate; RMSSD, Root Mean Square of Successive Differences.

-*n=95, **n=97, †n= 98, ‡n= 99.
